# Circulating Vitamin D Levels and DNA Repair Capacity in Four Molecular Subtypes of Women with Breast Cancer

**DOI:** 10.3390/ijms21186880

**Published:** 2020-09-19

**Authors:** Carmen Ortiz-Sánchez, Jarline Encarnación-Medina, Ralphdy Vergne, Luis Padilla, Jaime Matta

**Affiliations:** 1 Department of Basic Sciences, Ponce Research Institute, Ponce Health Sciences University-School of Medicine, Ponce, PR 00716, USA; jencarnacion@psm.edu (J.E.-M.); jmatta@psm.edu (J.M.); 2 Biomedical Sciences Department, Interamerican University of Puerto Rico, Ponce, PR 00715, USA; ralphdy.vergne@upr.edu; 3 Biology Department, University of Puerto Rico at Ponce, Ponce, PR 00716, USA; luis.padilla7@upr.edu

**Keywords:** plasma 25-hydroxyvitamin D (25(OH)D), vitamin D, breast cancer, DNA repair capacity, host-cell reaction assay, nucleotide excision repair, molecular subtypes

## Abstract

Vitamin D regulates estrogen synthesis among other mechanisms involved in breast cancer (BC) development; however, no evidence has been found regarding its relationship with DNA repair capacity (DRC). Therefore, the objective of this study was to elucidate whether DRC levels are linked with plasma 25(OH)D levels. BC cases and controls were selected from our BC cohort. DRC levels were assessed in lymphocytes through the host-cell reactivation assay. 25(OH)D levels were measured using the UniCel DxI 600 Access Immunoassay System. BC cases (*n* = 91) showed higher 25(OH)D levels than the controls (*n* = 92) (*p* = 0.001). When stratifying BC cases and controls into low and high DRC categories, BC cases with low DRC (*n* = 74) had the highest 25(OH)D levels (*p* = 0.0001). A positive correlation between 25(OH)D and DRC levels was found for the controls (*r* = 0.215, *p* = 0.043) while a negative correlation was found for BC cases (*r* = −0.236, *p* = 0.026). Significant differences in 25(OH)D levels were observed when stratifying by molecular subtypes (*p* = 0.0025). Our study provides evidence of a link between 25(OH)D and DRC in BC along with a description of to how 25(OH)D levels vary across subtypes. The positive correlation observed in the control group suggests that 25(OH)D contributes differently to DRC levels once the malignancy is developed.

## 1. Introduction

Worldwide, breast cancer (BC) accounts for nearly a quarter of all cancers in women [1]. About 1 in 8 women are expected to be diagnosed with BC. This accounts for 268,600 new cases of invasive BC in women in the U.S. only, including Hispanics [2,3]. For the last three decades, the use of vitamins, multivitamins, and supplements (i.e., calcium) has been attractive for BC prevention or as adjuvant treatment for this disease [4,5]. Vitamin D has different roles in cancer and healthy states. Previous studies have shown that vitamin D plays an important role in preventing the tumor initiation through anti-inflammatory and antioxidant defense mechanisms and DNA damage repair processes [6]. This highlights the role of vitamin D in cancer prevention. However, inconsistencies in study results hinder a clear understanding about whether vitamin supplementation could be beneficial once BC is developed [7,8]. A case–control study by Vergne et al., 2013 suggests that multivitamin supplementation could be an independent protective factor for BC. However, when other individual vitamins were taken into consideration (i.e., vitamins A, E, and C), no positive results were found. Interestingly, this study also found an association between calcium intake and having high overall DNA repair capacity (DRC) levels measured through the nucleotide excision repair (NER) pathway [9]. This pathway is one of the various DNA repair mechanisms responsible for maintaining the genomic stability and preventing alterations to the DNA that could potentially lead to cancer [10]. Therefore, it is not surprising that defective DNA repair, measured in lymphocytes, has been identified as a risk factor for different types of cancer [11,12,13], including BC [14]. Variability on DRC levels has been reported among the four principal molecular BC subtypes, where the triple-negative subtype showed the lowest levels [15].

Breast tumors may (+) or may not (−) have three hormonal receptors: estrogen (ER), progesterone (PR), and HER2 (human epidermal growth factor receptor 2). Based on their hormonal receptor status, four principal molecular BC subtypes have been identified: luminal A (ER+, PR+, HER2−), luminal B (ER+, PR+, HER2+), HER2+ (ER−, PR−, HER2+), and triple-negative (TN) (ER−, PR−, HER2−). Various studies have aimed at elucidating the role of vitamin D (25-hydroxyvitamin D (25(OH)D)) in BC [16,17,18] and more specifically among molecular subtypes. Low serum 25(OH)D levels have been associated with aggressive phenotypes and worse prognosis in several molecular BC subtypes [19].

A substantial amount of studies have shown discrepancies regarding vitamin D supplementation and its role in BC based on in vitro [20,21], in vivo [22,23], and clinical studies [18,24]. Other studies have reported an association between higher 25(OH)D levels and a lower BC risk but these findings have been inconsistent [8,25]. As part of its many physiological functions, vitamin D signaling influences estrogen synthesis. Since BC is known to be a hormonal cancer, due to its dependence on estrogen to promote cell proliferation [26], there is a need to understand the effects of vitamin D in women with BC [27,28]. Since previous studies from our laboratory established an association between DRC levels and ER positivity in women with BC, the main aim of this study was to elucidate whether DRC is linked with plasma 25(OH)D levels. A secondary aim was to understand whether this effect can be observed across the four principal BC subtypes. In general, we expected to observe differences in vitamin D levels between women with and without BC. We hypothesize that 25(OH)D levels will vary among women with different molecular BC subtypes and that women with ER+ breast tumors will present distinct plasma vitamin D levels when compared to women with ER− tumors.

## 2. Results

### 2.1. Plasma 25(OH)D Levels

Overall, BC cases included women in the age range from 41 to 60 years of age with high DRC levels, and a BMI over 25 kg/m^2^ who were not undergoing menopause. Controls were similar to BC cases regarding age and BMI (Table 1). In terms of DRC levels, most of the BC cases had low DRC levels using the previously established cut-off of low (<3.8%) and high DRC (≥3.8%) levels [29]. Controls were equally distributed into low and high DRC levels. For all women, the season of blood collection was recorded and no significant differences were observed.

Crude and adjusted analyses of plasma 25(OH)D levels were performed between BC cases and controls. Adjustments were performed by age and BMI since these two variables are known to influence the measurement of vitamin D in plasma [30]. Overall, BC cases (*n* = 91) showed higher levels of 25(OH)D when compared to the controls (*n* = 92) (Table 2). This difference was significant only after adjustment, where BC cases had 40.82 ± 1.03 ng/mL and controls had 38.55 ± 1.03 ng/mL (*p* = 0.001). When stratifying BC cases and controls into low and high DRC categories, significant differences in 25(OH)D levels were found on the crude and adjusted analyses (*p* = 0.0001) (Table 2). BC cases with low DRC (*n* = 74) had the highest mean levels with 42.36 ± 1.10 ng/mL followed by controls with high DRC (41.27 ± 1.41 ng/mL, *n* = 46). Mean plasma 25(OH)D levels for controls with low DRC levels (*n* = 46) were 35.74 ± 1.40 ng/mL, while BC cases with high DRC levels (*n* = 17) had 34.59 ± 2.30 ng/mL.

Pairwise comparisons were performed to evaluate the mean differences among groups (Table 3). Controls with high DRC levels had 5.54 ng/mL above the mean 25(OH)D levels when compared to controls with low DRC levels (*p* < 0.05). The mean 25(OH)D levels for BC cases with high DRC levels was 7.77 ng/mL less than BC cases with low DRC levels (*p* < 0.05). Cases with high DRC levels had lower 25(OH)D levels when compared to the controls independently of DRC levels. However, significant differences were found when comparing BC cases high DRC with controls also with high DRC levels (*p* < 0.05).

### 2.2. Correlation between 25(OH)D and DRC Levels

**Figure 1:**
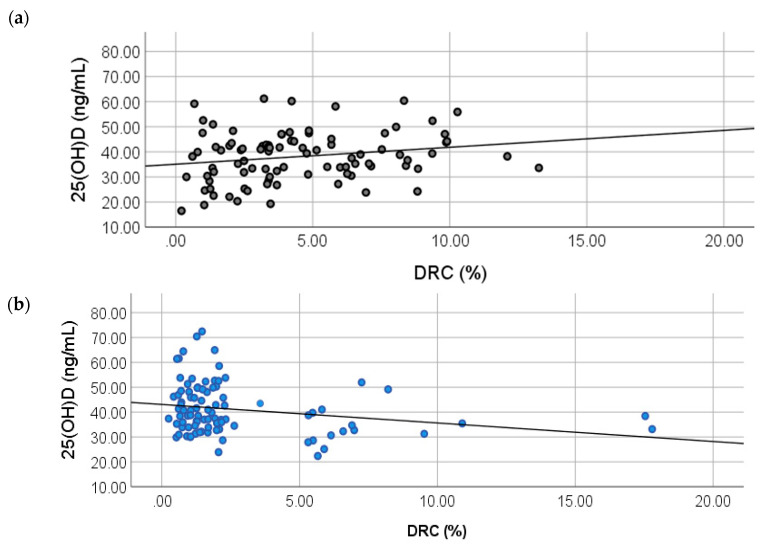
Correlations between DNA repair capacity (DRC) and 25(OH)D levels in breast cancer cases and controls. Partial correlation analyses were performed to test any correlations between DRC (%) and plasma vitamin D levels in (**a**) controls (*r* = 0.0215, *p* = 0.043) and (**b**) BC cases (*r* = −0.236; *p* = 0.026).

To study the relationship between 25(OH)D levels and DRC, crude and adjusted partial lineal correlation analyses were performed (Table 4). The partial correlation Model 1 shows a positive correlation of 0.215 for the controls (Figure 1A) (*p* = 0.040). For the BC cases group (model 2), a negative correlation was found with a coefficient of −0.236 (*p* = 0.030) (Figure 1B). After adjusting for confounders in both models, a statistical significance was still detected, showing a relationship between DRC and 25(OH)D levels (Table 4).

### 2.3. Relationship between Molecular Breast Cancer Subtypes and 25(OH)D Levels

To further elucidate the relevance of our results, additional analyses were performed to evaluate whether plasma 25(OH)D levels vary depending on the molecular subtype of the tumor. Since previous studies from our research team showed that the DRC level distribution is positively skewed in BC cases [15], we focused our analysis on BC cases with low DRC levels only. Mean 25(OH)D levels were compared among women with breast tumors that were classified as luminal A, luminal B, HER2+, and TN. Controls were also included on these analyses.

Crude analysis showed significant differences in 25(OH)D levels when stratifying by molecular subtypes: Luminal A (*n* = 17), Luminal B (*n* = 11), HER2+ (*n* = 9), and TN (*n* = 17); and comparing with low DRC controls (*n* =46) (*p* = 0.0025) (Table 5). Statistical significance increased after adjustment by age and BMI (*p* = 0.001), BC cases with HER2+ tumors showed the highest 25(OH)D levels (47.70 ± 3.14 ng/mL) followed by BC cases with TN tumors (45.08 ± 2.24 ng/mL). Luminal B BC cases had a mean 25(OH)D concentration of 40.50 ± 2.78 ng/mL while luminal A BC cases had 38.73 ± 2.26 ng/mL. Controls showed the lowest 25(OH)D levels with 35.51 ± 1.36 ng/mL.

When exploring the mean differences in 25(OH)D levels among groups, significant differences were observed between controls and cases with HER2+ and TN BC. Controls had 12.19 ng/mL and 9.57 ng/mL less than HER2+ (*p* = 0.001) and TN (*p* = 0.0001) BC cases, respectively (Table 6). BC cases with luminal A tumors had 8.98 ng/mL less 25(OH)D levels than HER2+ BC cases (*p* = 0.025). Therefore, our results show that vitamin D levels vary among molecular BC subtypes. In addition, our results show that ER negative subtypes have the highest 25(OH)D levels.

## 3. Discussion

Vitamin D is a molecule with pleiotropic functions. This study adds a new functional dimension to 25(OH)D, the major circulating form of vitamin D, namely, the link between its plasma levels and DRC levels in women with BC. This study also presents the differences in 25(OH)D levels across the four principal molecular BC subtypes in Puerto Rican women. Our study is the first, to our knowledge, in aiming to elucidate the relationship between 25(OH)D and DRC using lymphocytes as surrogate markers for the participants’ DRC specifically through the NER pathway. Interestingly, our results show plasma 25(OH)D levels vary significantly in women with and without BC. Moreover, BC cases with low DRC levels had higher levels of 25(OH)D when compared to BC cases with high DRC levels. This finding is further confirmed by the negative partial correlation found between DRC and 25(OH)D levels in BC cases. Of the relationship between plasma 25(OH)D levels and DNA repair, little is known. A study by Gonzalez-Suarez et al. (2011) provided the first evidence of a link between vitamin D and double-strand break DNA repair [31], showing that calcitriol (biologically-active form of vitamin D) could stabilize 53BP1 (p53 binding protein 1) levels in human cells. 53BP1 has been recently highlighted as a novel target for BRCA1-deficient TN BC [32]. Moreover, Gonzalo (2014) presented a novel sensitization strategy for TN BC by taking advantage of the calcitriol-mediated 53BP1 stabilization [32].

As for DNA damage, Wang et al. (2016) evaluated the connection between oxidative damage and vitamin D levels in a group of non-obese, non-smoking young adults; however, no association was found [33]. Interestingly, our results show a positive correlation between 25(OH)D levels and DRC levels for the controls. This could support our hypothesis that 25(OH)D levels change once the malignancy is developed. Moreover, adding to the benefits of maintaining optimal levels of vitamin D. As of today, no studies linking single-strand break repair and 25(OH)D have been performed.

Our results show a significant difference in vitamin D levels between treatment-naïve BC cases and controls at the time of diagnosis. Previous studies report different outcomes for this comparison such as the study by Abbas et al. (2009) in a German BC case–control cohort, in which plasma 25(OH)D levels were 18.2 ng/mL and 20.5 ng/mL for cases and controls, respectively. Although the BC cases were described as recently diagnosed, the difference between time of diagnosis and blood collection was 189 days [34]. Similar trends have been reported by several groups [16,19,35,36]; however, we have identified several factors that could potentially explain our results. First, most of the studies regarding 25(OH)D and BC have been performed in White women with none (or a limited number) considering Hispanics [16,19,24,37]. This is important since previous studies have documented that ethnicity or race may influence the association between vitamin D levels and BC risk [38]. Since UV radiation is the main source for vitamin D synthesis, the geographical location of the study population is crucial in order to establish comparisons among studies. Although most of the vitamin D studies in BC have been performed in the US, other studies have been performed in Korean [38], German [34], Japanese [39], and French [35] women cohorts. As of today, few studies have been performed in Hispanic populations [40,41] and none including Puerto Rican women with BC. Few studies had been performed to measure the plasma vitamin D levels in the Puerto Rican population [42,43,44]; however, the largest study was performed by Suárez-Martínez et al. (2013). In this study, the laboratory test results of 4090 individuals who had vitamin D levels measured at a reference laboratory located in Puerto Rico’s metropolitan area were analyzed. This group reported that around 32% of the studied population was vitamin D sufficient (>30 ng/mL) [42]. Of this study group 3414 of the participants were women with a mean vitamin D concentration of 26.9 ± 12.3 ng/mL. Although the Suárez-Martinez’s study provides the first report of plasma vitamin D levels in Puerto Rico, the lack of relevant participants’ information does not allow for a clear comparison with our study. First, no exclusion criteria were established. No medical information was collected; therefore, there is no assurance of the health status of the study participants. In addition, no epidemiological information was collected and only six municipalities (out of 78) of are represented by their findings whereas our study includes participants from almost 36 municipalities.

Another important consideration is the timing of blood sample collection in relation to BC diagnosis [37]. In most of the studies, the time between blood collection and diagnosis ranges between 2 months and years [16,37,45,46]. In our study, the blood collection was performed at the time of diagnosis which allowed us to avoid any confounding effects due to BC treatment. As previously reported, BC therapy can alter plasma vitamin D levels [47,48]. It has been reported that after 6 months of chemotherapy, vitamin D levels can be reduced to almost 5.52 ng/mL (*p* = 0.003). In contrast, patients receiving anti-hormone therapy showed an increase in vitamin D levels at 6 months and 12 months (+3.00 ng/mL and +6.47 ng/mL, respectively) [47].

Our results show that plasma 25(OH)D levels are significantly different across the four principal molecular BC subtypes, with ER negative subtypes having the highest levels. The work of Peppone et al. (2012) is one of the few studies that aimed to elucidate the relationship of plasma vitamin D among molecular BC subtypes. This group found that women with basal-like tumors had lower vitamin D levels than women with luminal A tumors with 24.2 ng/mL and 32.8 ng/mL, respectively (*p* = 0.04) [19]. In contrast with our findings, this group found that women with ER+ tumors had higher vitamin D levels than women with ER− tumors. This group also found significant differences in plasma vitamin D levels among the four molecular subtypes; however, in contrast with our findings, they found that ER− subtypes had the lowest vitamin D concentrations [19].

This study provides the first evidence of a link between plasma 25(OH)D and DRC levels in Hispanic women with and without BC. This study also shows the variation in 25(OH)D levels across the four principal molecular BC subtypes in this population. Moreover, the positive correlation observed in the control group suggests that circulating 25(OH)D contributes differently to the DRC levels once the malignancy is developed. This finding adds to the known benefits of maintaining optimal vitamin D levels in women without BC. Since having a low DRC level has been associated with an increased BC risk, maintaining optimal vitamin D levels could be viewed as a potential tool for chemoprevention. On the other hand, once the malignancy is developed, our results show that vitamin D levels become altered and that there are variations among molecular subtypes. Various studies suggest that a different approach regarding vitamin use is recommended for cancer patients. A recent study by Ambrose et al. (2020), highlights that the use of multivitamin and antioxidants before and during chemotherapy could have a negative impact on recurrence and overall survival in women with BC [49]. Therefore, our study adds to the overall knowledge regarding vitamin D levels in recently diagnosed treatment-naïve women with BC. Our results also provide new insights on the role of 25(OH)D in DRC levels in women with BC while opening new avenues for mechanistic studies to study this effect.

## 4. Materials and Methods

### 4.1. Patient Recruitment

This study was approved by the Ponce Health Sciences University Institutional Review Board (IRB #120207-JM renewed on 24 October 2019). Informed consent and blood samples were collected by the study nurse along with epidemiological data through a questionnaire. Participants were selected from our BC cohort of Puerto Rican women recruited from 2006 to 2013 [50]. Women without BC (controls) were required to have a normal breast examination performed by a primary care physician and a normal mammography 6 months prior to study enrolment. The inclusion criteria for women with BC (cases) were recently diagnosed, treatment-naïve (had not received blood transfusions, chemotherapy, or radiotherapy) patients with primary breast tumors. Pathology reports from BC cases were obtained to confirm the diagnosis, and collect clinicopathological variables such as: tumor grade, tumor size, and other clinically relevant information. For this study, we established an exclusion criteria based on previous studies including: intake of vitamin D, vitamin E, vitamin C, calcium, steroids, cortisone, hormone replacement therapy [51], insulin, or any immune repressors [52].

### 4.2. Hormone Receptor Status

Medical records from women with BC included in this study were reviewed to collect receptor status data (ER, PR, and HER2). Receptor status was assessed on the participants’ formalin-fixed tumors using immunohistochemistry (IHC) according to ASCO (American Society of Clinical Oncology) and CAP (College of American Pathologists) guidelines [53,54] in 10 private laboratories in Puerto Rico. Information regarding ER and PR status included: percentage of positive-staining cells, intensity of staining (weak, moderate, or strong), and a result interpretation based on the percentage of invasive tumor cells that were positively stained for ER/PR (where ≥1% meant “receptor positive” and <1% meant “receptor negative”) [54]. HER2 testing was performed using FDA-approved IHC assays followed by Fluorescence In Situ Hybridization (FISH) if equivocal results were obtained (2+ or 1+) [54]. For our analysis, HER2 status was categorized as a dichotomous variable: “positive” (3+) or “negative” (2+ to 0).

### 4.3. DNA Repair Capacity (DRC) Measurements

DRC was assessed in the participants’ lymphocytes through the host-cell reactivation (HCR) assay with a luciferase reporter gene, as previously published [50]. Lymphocytes were used as surrogate markers of the participants’ DRC [55,56]. At the moment of recruitment, peripheral blood samples (30 mL) were collected in heparinized tubes from each participant to obtain lymphocytes that were assayed in batches. Lymphocytes with >95% viability were incubated for 72 h with phytohemagglutinin and then transfected with undamaged or UVC light damaged plasmid DNA. After transfection, repair-transcription-blocking damage was introduced exogenously on foreign DNA; then DRC was measured via HCR [57]. The HCR allows for a direct measurement of in vivo DRC. This approach measured the unaffected phenotype, which reflects the cells’ inherent DRC, measured primarily in terms of their NER activity [57]. Cells isolated from Xeroderma pigmentosum patients corresponding to complementation groups C and D were used as internal controls (GM02246D and GM02253F, respectively; Coriell Institute Medical Research; Camden, NJ, USA).

### 4.4. Calculation of DRC

Gene expression of luciferase activity was measured using a luminometer (Turner Designs, model TD-20/20, Sunnyvale, CA, USA). DRC was calculated as the percentage of luciferase activity present after damaged plasmid DNA repair, compared to the undamaged plasmid DNA repair (100%). Results were expressed as percentage of residual luciferase reporter gene expression (% luciferase activity in luminescence units).

### 4.5. Plasma 25-Hydroxyvitamin D Levels

Plasma samples obtained from peripheral blood were stored in aliquots (−80 °C) until vitamin D levels were measured in a single batch in October 2017. Vitamin D levels were measured using the UniCel DxI 600 Access Immunoassay System (Beckman Coulter, CA, USA) through the Access 25(OH) Vitamin D Total Assay which detects both 25(OH)vitamin D2 and 25(OH)vitamin D3. For this study, we used the 25(OH)D as an indicator of vitamin D levels in plasma. The analysis was performed at Laboratorios Ramírez, a CLIA-certified laboratory located in Ponce, Puerto Rico.

### 4.6. Statistical Analyses

Demographic and clinicopathological variables were analyzed using contingency tables and chi-square tests. Yates’ correction was applied when the number of observations was less than 5. Mean 25(OH)D comparisons between and among groups were performed using an independent t test and analysis of covariance (ANCOVA) adjusting for potential cofounders including age and BMI. Pairwise comparisons were performed to detect differences in 25(OH)D after adjustment for age and BMI. To assess the relationship of the DRC on the 25(OH)D levels, partial lineal correlation analyses were performed also adjusting by age and BMI. Significance levels were established using a *p*-value cutoff of 0.05 based on a two-tail test. The data were analyzed using SPSS 25.0 software (Chicago, IL, USA).

## Figures and Tables

**Table 1:** Description of the study population including women with and without breast cancer.

Variables	Controls	BC Cases	*p*-Value ^1^
	*n* = 92 (50.27%)	*n* = 91 (49.73%)	
DRC			<0.0001
Low (<3.8%)	46 (25.13)	74 (40.44)	
High (≥3.8%)	46 (25.14)	17 (9.29)	
Age			0.6520
21–40	23 (12.57)	19 (10.38)	
41–60	54 (29.51)	53 (28.96)	
61+	15 (8.20)	19 (10.38)	
BMI			1.0000
<25 kg/m^2^	31 (16.94)	32 (17.49)	
≥25 kg/m^2^	60 (32.79)	59 (32.24)	
Missing	1 (0.55)	-	
Ever been pregnant			0.6775
Yes	80 (43.72)	77 (42.08)	
No	12 (6.56)	14 (7.65)	
Ever breastfeed			0.0528
Yes	47 (25.68)	32 (17.49)	
No	32 (17.49)	42 (22.95)	
Missing	13 (7.10)	17 (9.29)	
Length of breastfeeding			0.0281
Never	45 (24.59)	59 (32.24)	
1–5 months	17 (9.29)	26 (14.21)	
≥6 months	27 (14.75)	6 (3.28)	
Missing	3 (1.64)	-	
Oral contraceptive use			1.0000
Yes	47 (25.68)	46 (25.14)	
No	45 (24.59)	44 (24.04)	
Missing	-	1 (0.55)	
Age started oral contraceptive			0.1446
<20	14 (7.65)	8 (4.37)	
≥21	29 (15.85)	36 (19.67)	
Missing	4 (2.19)	2 (1.09)	
Menopause			0.8441
Yes	15 (8.20)	16 (8.74)	
No	75 (40.98)	71 (38.80)	
Missing	2 (1.09)	4 (2.19)	
BC history in any family member			0.2709
Yes	26 (14.21)	33 (18.03)	
No	66 (36.07)	58 (31.69)	
Family history of cancer (not BC)			0.2850
Yes	54 (29.51)	61 (33.33)	
No	38 (20.77)	30 (16.39)	
Season of blood collection			0.6768
Spring (Feb–Apr)	30 (16.39)	23 (12.57)	
Summer (May–Jul)	19 (10.38)	24 (13.11)	
Fall (Aug–Oct)	21 (11.48)	22 (12.02)	
Winter (Nov–Jan)	22 (12.02)	22 (12.02)	

^1^*p*-value was obtained from Chi-squared and Fisher’s exact test. BC: breast cancer.

**Table 2:** Mean 25(OH)D level comparisons among women with and without breast cancer with low and high DNA repair capacity.

Stratifications	25(OH)D (ng/mL) (Mean ± SD) ^a^	25(OH)D (ng/mL) (Mean ± SD) *	*p*-Value	95% Confidence Interval	
				Lower Bound	Upper Bound
Controls	38.10 (9.95)	38.55 (1.03)	0.123 ^a^, 0.001 *	36.51	40.59
BC Cases	41.12 (10.25)	40.82 (1.03)		38.79	42.85
Controls LDRC	35.41 (10.46)	35.74(1.40)	0.0001 ^a^, 0.0001*	32.98	38.43
Controls HDRC	41.18 (8.43)	41.27 (1.41)		38.49	44.05
BC Cases LDRC	42.55 (10.25)	42.36 (1.10)		40.18	44.53
BC Cases HDRC	34.90 (7.78)	34.59 (2.30)		30.06	39.13

^a^ Crude mean comparisons; * adjusted by age and BMI. BC: breast cancer; LDRC: low DNA repair capacity; HDRC: high DNA repair capacity.

**Table 3:** Differences in mean 25(OH)D levels among women with and without breast cancer with low and high DNA repair capacity.

Stratifications		Mean 25(OH)D Difference (ng/mL)	Significance ^b^	95% Confidence Interval for Difference ^b^	
				Lower Bound	Upper Bound
Controls LDRC	Controls HDRC	−5.54 *	0.006	−9.45	−1.63
	BC cases LDRC	−6.62 *	0.0001	−10.14	−3.10
	BC cases HDRC	1.14	0.671	−4.17	6.45
Controls HDRC	Controls LDRC	5.54 *	0.006	1.63	9.45
	BC cases LDRC	−1.09	0.545	−4.62	2.45
	BC cases HDRC	6.68 *	0.014	1.36	12.00
BC cases LDRC	Controls LDRC	6.62 *	0.0001	3.10	10.14
	Controls HDRC	1.09	0.545	−2.45	4.62
	BC cases HDRC	7.77 *	0.003	2.74	12.80
BC cases HDRC	Controls LDRC	−1.14	0.617	−6.45	4.17
	Controls HDRC	−6.68 *	0.014	−12.00	−1.36
	BC cases LDRC	−7.77 *	0.003	−12.80	−2.74

Based on estimated marginal means. * 25(OH)D mean difference is significant at the 0.05 level; ^b^ adjustment for multiple comparisons: Least Significant Difference (equivalent to no adjustments). BC: breast cancer; LDRC: low DNA repair capacity; HDRC: high DNA repair capacity.

**Table 4:** Correlation analyses between plasma 25(OH)D and DNA repair capacity levels in women with and without breast cancer.

Stratifications	R^2^		Testing Significance	
	Model 1	Model 2	*p*-Value ^a^	*p*-Value *
Controls	0.215	-	0.040	0.043
BC Cases	-	−0.236	0.030	0.026

^a^ Crude mean comparisons; * adjusted by age and BMI. BC: breast cancer.

**Table 5:** Mean 25(OH)D level comparisons among women with low DNA repair capacity levels stratified by molecular subtypes.

Stratifications	25(OH)D (ng/mL) (Mean ± SD) ^a^	25(OH)D (ng/mL) (Mean ± SD) *	*p*-Value	95% Confidence Interval	
				Lower Bound	Upper Bound
Controls	38.41 (10.46)	35.51 (1.36)	0.0025 ^a^, 0.001 *	32.81	38.211
Luminal A	38.46 (6.79)	38.73 (2.26)		34.23	43.22
Luminal B	40.99 (8.22)	40.50(2.78)		34.98	46.03
HER2+	48.83 (11.34)	47.70 (3.14)		41.46	53.94
Triple-negative	44.71 (10.26)	45.08 (2.24)		40.62	49.53

^a^ Crude mean comparisons; * adjusted by age and BMI.

**Table 6:** Differences in mean 25(OH)D levels among women with BC with low DRC with different molecular subtypes.

Stratifications		Mean 25(OH)D Difference	Significance ^b^	95% Confidence Interval for Difference ^b^	
				Lower Bound	Upper Bound
Controls	Luminal A	3.21	0.225	−8.45	2.01
	Luminal B	−4.99	0.110	−11.14	1.16
	HER2+	−12.19 *	0.001	−19.01	−5.38
	TN	−9.57 *	0.0001	−14.78	−4.36
Luminal A	Controls	3.21	0.225	−2.02	8.45
	Luminal B	−1.78	0.621	−8.90	5.34
	HER2+	−8.98 *	0.025	−16.78	−1.18
	TN	−6.35	0.050	−12.71	0.002
Luminal B	Controls	4.99	0.110	−1.16	11.14
	Luminal A	1.78	0.621	−5.34	8.90
	HER2+	−7.20	0.089	−15.52	1.13
	TN	−4.58	0.204	−11.68	2.53
HER2+	Controls	12.19 *	0.001	5.38	19.05
	Luminal A	8.98 *	0.025	1.18	16.78
	Luminal B	7.20	0.089	−1.13	15.53
	TN	2.63	0.497	−5.01	10.26
TN	Controls	9.57 *	0.0001	4.36	14.78
	Luminal A	6.35	0.050	−0.002	12.71
	Luminal B	4.58	0.204	−2.53	11.68
	HER2+	−2.63	0.497	−10.26	5.01

Based on estimated marginal means. * 25(OH)D mean difference is significant at the 0.05 level; ^b^ adjustment for multiple comparisons: Least Significant Difference (equivalent to no adjustments).

## Abbreviations

BC	Breast cancer
DRC	DNA repair capacity
LDRC	Low DRC
HDRC	High DRC
NER	Nucleotide excision repair
ER	Estrogen
PR	Progesterone
HER2	Human epidermal growth factor receptor 2
TN	Triple-negative
25(OH)D	25-hydroxyvitamin D
BMI	Body mass index
UV	Ultraviolet
IRB	Institutional Review Board
IHC	Immunohistochemistry
ASCO	American Society of Clinical Oncology
CAP	College of American Pathologists
FDA	Food and Drug Administration
FISH	Fluorescence In Situ Hybridization
HCR	Host-cell reactivation
CLIA	Clinical Laboratory Improvement Amendments
ANCOVA	Analysis of covariance

## References

[1] Ferlay J., Soerjomataram I., Ervik M., Dikshit R., Eser S., Mathers C., Rebelo M., Parkin D.M., Forman D., Bray F. (2013). GLOBOCAN 2012 v1.0, Cancer Incidence and Mortality Worldwide: IARC CancerBase No. 11.

[2] American Cancer Society (2019). Breast Cancer Facts & Figures 2019–2020.

[3] Martinez Tyson D., Medina-Ramirez P., Flores A.M., Siegel R., Aguado Loi C. (2018). Unpacking Hispanic Ethnicity—Cancer Mortality Differentials among Hispanic Subgroups in the United States, 2004–2014. Front. Public Health.

[4] Yasueda A., Urushima H., Ito T. (2016). Efficacy and Interaction of Antioxidant Supplements as Adjuvant Therapy in Cancer Treatment: A Systematic Review. Integr. Cancer Ther..

[5] Norman H.A., Butrum R.R., Feldman E., Heber D., Nixon D., Picciano M.F., Rivlin R., Simopoulos A., Wargovich M.J., Weisburger E.K. (2003). The Role of Dietary Supplements during Cancer Therapy. J. Nutr..

[6] Jeon S.-M., Shin E.-A. (2018). Exploring vitamin D metabolism and function in cancer. Exp. Mol. Med..

[7] Kennel K.A., Drake M.T. (2013). Vitamin D in the cancer patient. Curr. Opin. Support. Palliat. Care.

[8] Crew K.D. (2013). Vitamin D: Are we ready to supplement for breast cancer prevention and treatment?. ISRN Oncol..

[9] Vergne Y., Matta J., Morales L., Vargas W., Alvarez-Garriga C., Bayona M. (2013). Breast Cancer and DNA Repair Capacity: Association with Use of Multivitamin and Calcium Supplements. Integr. Med..

[10] Marteijn J.A., Lans H., Vermeulen W., Hoeijmakers J.H. (2014). Understanding nucleotide excision repair and its roles in cancer and ageing. Nat. Rev. Mol. Cell Biol..

[11] Wei Q., Cheng L., Hong W.K., Spitz M.R. (1996). Reduced DNA repair capacity in lung cancer patients. Cancer Res..

[12] Wei Q., Matanoski G.M., Farmer E.R., Hedayati M.A., Grossman L. (1993). DNA repair and aging in basal cell carcinoma: A molecular epidemiology study. Proc. Natl. Acad. Sci. USA.

[13] Hu J.J., Hall M.C., Grossman L., Hedayati M., McCullough D.L., Lohman K., Case L.D. (2004). Deficient Nucleotide Excision Repair Capacity Enhances Human Prostate Cancer Risk. Cancer Res..

[14] Ramos J.M., Ruiz A., Colen R., Lopez I.D., Grossman L., Matta J.L. (2004). DNA repair and breast carcinoma susceptibility in women. Cancer.

[15] Matta J., Ortiz C., Encarnacion J., Dutil J., Suarez E. (2017). Variability in DNA Repair Capacity Levels among Molecular Breast Cancer Subtypes: Triple Negative Breast Cancer Shows Lowest Repair. Int. J. Mol. Sci..

[16] McCullough M.L., Stevens V.L., Patel R., Jacobs E.J., Bain E.B., Horst R.L., Gapstur S.M., Thun M.J., Calle E.E. (2009). Serum 25-hydroxyvitamin D concentrations and postmenopausal breast cancer risk: A nested case control study in the Cancer Prevention Study-II Nutrition Cohort. Breast Cancer Res..

[17] Deeb K.K., Trump D.L., Johnson C.S. (2007). Vitamin D signalling pathways in cancer: Potential for anticancer therapeutics. Nat. Rev. Cancer.

[18] Palmieri C., MacGregor T., Girgis S., Vigushin D. (2006). Serum 25-hydroxyvitamin D levels in early and advanced breast cancer. J. Clin. Pathol..

[19] Peppone L.J., Rickles A.S., Janelsins M.C., Insalaco M.R., Skinner K.S. (2012). The Association between Breast Cancer Prognostic Indicators and Serum 25-OH Vitamin D Levels. Ann. Surg. Oncol..

[20] Swami S., Raghavachari N., Muller U.R., Bao Y.P., Feldman D. (2003). Vitamin D Growth Inhibition of Breast Cancer Cells: Gene Expression Patterns Assessed by cDNA Microarray. Breast Cancer Res. Treat..

[21] James S.Y., Mackay A.G., Colston K.W. (1996). Effects of 1,25 dihydroxyvitamin D3 and its analogues on induction of apoptosis in breast cancer cells. J. Steroid Biochem. Mol. Biol..

[22] Matthews D., LaPorta E., Zinser G.M., Narvaez C.J., Welsh J. (2010). Genomic Vitamin D signaling in Breast Cancer: Insights from Animal Models and Human Cells. J. Steroid Biochem. Mol. Biol..

[23] Welsh J. (2004). Vitamin D and breast cancer: Insights from animal models. Am. J. Clin. Nutr..

[24] Crew K.D., Gammon M.D., Steck S.E., Hershman D.L., Cremers S., Dworakowski E., Shane E., Terry M.B., Desai M., Teitelbaum S.L. (2009). Association between Plasma 25-Hydroxyvitamin D and Breast Cancer Risk. Cancer Prev. Res..

[25] Goodwin P.J., Ennis M., Pritchard K.I., Koo J., Hood N. (2009). Prognostic effects of 25-hydroxyvitamin D levels in early breast cancer. J. Clin. Oncol..

[26] Lumachi F., Santeufemia D.A., Basso S.M. (2015). Current medical treatment of estrogen receptor-positive breast cancer. World J. Biol. Chem..

[27] Lisse T.S., Hewison M., Adams J.S. (2011). Hormone response element binding proteins: Novel regulators of vitamin D and estrogen signaling. Steroids.

[28] Santos-Martinez N., Diaz L., Ordaz-Rosado D., Garcia-Quiroz J., Barrera D., Avila E., Halhali A., Medina-Franco H., Ibarra-Sanchez M.J., Esparza-Lopez J. (2014). Calcitriol restores antiestrogen responsiveness in estrogen receptor negative breast cancer cells: A potential new therapeutic approach. BMC Cancer.

[29] Encarnacion J., Ortiz C., Vergne R., Vargas W., Coppola D., Matta J.L. (2016). High DRC Levels Are Associated with Let-7b Overexpression in Women with Breast Cancer. Int. J. Mol. Sci..

[30] Han J., Guo X., Yu X., Liu S., Cui X., Zhang B., Liang H. (2019). 25-Hydroxyvitamin D and Total Cancer Incidence and Mortality: A Meta-Analysis of Prospective Cohort Studies. Nutrients.

[31] Gonzalez-Suarez I., Redwood A.B., Grotsky D.A., Neumann M.A., Cheng E.H., Stewart C.L., Dusso A., Gonzalo S. (2011). A new pathway that regulates 53BP1 stability implicates cathepsin L and vitamin D in DNA repair. EMBO J..

[32] Gonzalo S. (2014). Novel roles of 1alpha,25(OH)2D3 on DNA repair provide new strategies for breast cancer treatment. J. Steroid Biochem. Mol. Biol..

[33] Wang E.W., Collins A.R., Pang M.Y.C., Siu P.P.M., Lai C.K.Y., Woo J., Benzie I.F.F. (2016). Vitamin D and oxidation-induced DNA damage: Is there a connection?. Mutagenesis.

[34] Abbas S., Chang-Claude J., Linseisen J. (2009). Plasma 25-hydroxyvitamin D and premenopausal breast cancer risk in a German case-control study. Int. J. Cancer.

[35] Engel P., Fagherazzi G., Boutten A., Dupré T., Mesrine S., Boutron-Ruault M.-C., Clavel-Chapelon F. (2010). Serum 25(OH) Vitamin D and Risk of Breast Cancer: A Nested Case-Control Study from the French E3N Cohort. Cancer Epidemiol. Biomark. Prev..

[36] Rejnmark L., Tietze A., Vestergaard P., Buhl L., Lehbrink M., Heickendorff L., Mosekilde L. (2009). Reduced Prediagnostic 25-Hydroxyvitamin D Levels in Women with Breast Cancer: A Nested Case-Control Study. Cancer Epidemiol. Biomark. Prev..

[37] Bertone-Johnson E.R., Chen W.Y., Holick M.F., Hollis B.W., Colditz G.A., Willett W.C., Hankinson S.E. (2005). Plasma 25-Hydroxyvitamin D and 1,25-Dihydroxyvitamin D and Risk of Breast Cancer. Cancer Epidemiol. Biomark. Prev..

[38] Kim H.J., Lee Y.M., Ko B.S., Lee J.W., Yu J.H., Son B.H., Gong G.-Y., Kim S.B., Ahn S.H. (2011). Vitamin D Deficiency is Correlated with Poor Outcomes in Patients with Luminal-type Breast Cancer. Ann. Surg. Oncol..

[39] Kawase T., Matsuo K., Suzuki T., Hirose K., Hosono S., Watanabe M., Inagaki M., Iwata H., Tanaka H., Tajima K. (2010). Association between vitamin D and calcium intake and breast cancer risk according to menopausal status and receptor status in Japan. Cancer Sci..

[40] Farrag S.E., Dwivedi A.K., Otoukesh S., Badri N.J., Sanchez L.A., Nahleh Z.A. (2017). Prevalence of Low Vitamin D in Patients with Breast Cancer in a Predominantly Hispanic Population at the American-Mexican Border. Nutr. Cancer.

[41] Wu Y., Sarkissyan M., Clayton S., Chlebowski R., Vadgama J.V. (2017). Association of Vitamin D3 Level with Breast Cancer Risk and Prognosis in African-American and Hispanic Women. Cancers.

[42] Suarez-Martinez E.B., Perez C.M., Cruz S.K., Khorsandi S., Chardon C., Ferder L. (2013). Importance of vitamin D and vitamin D levels status in Puerto Ricans. J. Health Care Poor Underserved.

[43] Palacios C., Gil K., Pérez C.M., Joshipura K. (2012). Determinants of Vitamin D Status among Overweight and Obese Puerto Rican Adults. Ann. Nutr. Metab..

[44] Dávila L.H., Rivera N.R., Valentin M.L., Haddock L., Martínez R.R., Bossolo A.G., Vick M.R. (2015). Prevalence of vitamin D insufficiency and deficiency among medical residents of the University Hospital in San Juan, Puerto Rico. Puerto Rico Health Sci. J..

[45] Janowsky E.C., Lester G.E., Weinberg C.R., Millikan R.C., Schildkraut J.M., Garrett P.A., Hulka B.S. (1999). Association between low levels of 1,25-dihydroxyvitamin D and breast cancer risk. Public Health Nutr..

[46] Yao S., Kwan M.L., Ergas I.J., Roh J.M., Cheng T.D., Hong C.C., McCann S.E., Tang L., Davis W., Liu S. (2017). Association of Serum Level of Vitamin D at Diagnosis with Breast Cancer Survival: A Case-Cohort Analysis in the Pathways Study. JAMA Oncol..

[47] Kim H.J., Koh B.S., Yu J.H., Lee J.W., Son B.H., Kim S.B., Ahn S.H. (2014). Changes in serum hydroxyvitamin D levels of breast cancer patients during tamoxifen treatment or chemotherapy in premenopausal breast cancer patients. Eur. J. Cancer.

[48] Kok D.E., van den Berg M.M.G.A., Posthuma L., van ’t Erve I., van Duijnhoven F.J.B., de Roos W.K., Grosfeld S., Los M., Sommeijer D.W., van Laarhoven H.W.M. (2019). Changes in Circulating Levels of 25-hydroxyvitamin D3 in Breast Cancer Patients Receiving Chemotherapy. Nutr. Cancer.

[49] Ambrosone C.B., Zirpoli G.R., Hutson A.D., McCann W.E., McCann S.E., Barlow W.E., Kelly K.M., Cannioto R., Sucheston-Campbell L.E., Hershman D.L. (2020). Dietary Supplement Use During Chemotherapy and Survival Outcomes of Patients with Breast Cancer Enrolled in a Cooperative Group Clinical Trial (SWOG S0221). J. Clin. Oncol..

[50] Matta J., Echenique M., Negron E., Morales L., Vargas W., Gaetan F.S., Lizardi E.R., Torres A., Rosado J.O., Bolanos G. (2012). The association of DNA Repair with breast cancer risk in women. A comparative observational study. BMC Cancer.

[51] Deng H.W., Li J., Li J.L., Johnson M., Gong G., Davis K.M., Recker R.R. (1998). Change of bone mass in postmenopausal Caucasian women with and without hormone replacement therapy is associated with vitamin D receptor and estrogen receptor genotypes. Hum. Genet..

[52] Yetley E.A. (2008). Assessing the vitamin D status of the US population. Am. J. Clin. Nutr..

[53] Wolff A.C., Hammond M.E., Hicks D.G., Dowsett M., McShane L.M., Allison K.H., Allred D.C., Bartlett J.M., Bilous M., Fitzgibbons P. (2014). Recommendations for human epidermal growth factor receptor 2 testing in breast cancer: American Society of Clinical Oncology/College of American Pathologists clinical practice guideline update. Arch. Pathol. Lab. Med..

[54] Hammond M.E., Hayes D.F., Wolff A.C., Mangu P.B., Temin S. (2010). American Society of Clinical Oncology/College of American Pathologists Guideline Recommendations for Immunohistochemical Testing of Estrogen and Progesterone Receptors in Breast Cancer. J. Oncol. Pract. Am. Soc. Clin. Oncol..

[55] Mendez P., Taron M., Moran T., Fernandez M.A., Requena G., Rosell R. (2011). A modified host-cell reactivation assay to quantify DNA repair capacity in cryopreserved peripheral lymphocytes. DNA Repair.

[56] Athas W.F., Hedayati M.A., Matanoski G.M., Farmer E.R., Grossman L. (1991). Development and field-test validation of an assay for DNA repair in circulating human lymphocytes. Cancer Res..

[57] Wang L., Wei Q., Shi Q., Guo Z., Qiao Y., Spitz M.R. (2007). A modified host-cell reactivation assay to measure repair of alkylating DNA damage for assessing risk of lung adenocarcinoma. Carcinogenesis.

